# Serum Interleukins as Potential Prognostic Biomarkers in HBV-Related Acute-on-Chronic Liver Failure

**DOI:** 10.1155/2022/7794890

**Published:** 2022-09-08

**Authors:** Lili Zhang, Jianhua Hu, Chunyan Gou, Hua Jin, Chun Zhang, Yang Liu, Yitong Wang, Xiaojun Wang

**Affiliations:** ^1^Integrated Traditional Chinese and Western Medicine Center, Beijing Youan Hospital, Capital Medical University, Beijing (100069), China; ^2^Department of Infectious Diseases, Guang'anmen Hospital, China Academy of Chinese Medical Sciences, Beijing (100053), China

## Abstract

Hepatitis B virus-related acute-on-chronic liver failure (HBV-ACLF) is relatively common in China and has complex pathogenesis, difficult clinical treatment, and poor prognosis. Immune status is an important factor affecting ACLF prognosis. Interleukins are a family of secreted lymphocyte factors that interact with a host of cell types including immune cells. These signaling molecules play important roles in transmitting information; regulating immune cells; mediating the activation, proliferation, and differentiation of T and B cells; and modulating inflammatory responses. Many studies have investigated the correlation between interleukin expression and the prognosis of HBV-ACLF. This review focuses on the potential use of interleukins as prognostic biomarkers in HBV-ACLF. References were mainly identified through PubMed and CNKI search, including relevant studies published until December 2021. We have summarized reports of several promising diagnostic interleukin biomarkers that predict susceptibility to HBV-ACLF. The use of biomarkers to understand early prognosis can help devise different therapeutic measures and improve patient survival. Ongoing research on prognostic biomarkers of HBV-ACLF is promising, and future preclinical and clinical studies are warranted.

## 1. Introduction

Hepatitis B virus-related acute-on-chronic liver failure (HBV-ACLF) is a kind of acute liver function deterioration of chronic liver disease with immune dysfunction and is the main form of chronic acute liver failure (ACLF) [1–3] and characterized by coagulation and severe jaundice, usually within 4 weeks of complicated with ascites and/or hepatic encephalopathy [4]. ACLF progressing rapidly is the feature of mortality rates as high as 50%-90% [5]. A retrospective cohort study by Xiao et al. found that patients treated with artificial liver support system (ALSS) had a higher short-term survival rate than those treated with standard medical therapy. Furthermore, different modalities of ALSS were associated with different outcomes, with patients receiving hybrid artificial liver support system having higher short-term survival than those receiving plasma exchange artificial liver support system based [6]. Early identification and aggressive intervention may improve the prognosis of these patients. Thus, seeking reliable prognostic markers and will have a high risk of mortality and distinguishing reversible patients after treatment will help reduce the mortality of HBV-ACLF [7].

Acute exacerbations in patients with compensated cirrhosis lead to multiple organ failure and high short-term mortality [8]. The death of patients with HBV-ACLF is often associated with extrahepatic organ failure. Extrahepatic organ failure often occurs concurrently with liver failure and is associated with severe systemic inflammation [9]. Therefore, the inflammatory response is of great importance in the pathogenesis of ACLF, and systemic inflammatory response is considered as a marker of ACLF [10, 11] and affects the prognosis of ACLF [11–14]. Recruitment and differentiation of immune cells at inflammatory sites are activated and promoted by proinflammatory cytokines [15]. Interleukins refer to cytokines interacting with leukocytes or immune cells, which participate in transmitting information, activating and regulating immune cells, and mediating inflammatory reactions like activation, proliferation, and differentiation of T cells and B cells [16]. Several previous studies have shown that interleukin level in vivo is correlated with the prognosis of ACLF. This paper will comprehensively explore the relationship between interleukin level and the prognosis of HBV-ACLF.

## 2. Interleukin-1

IL-1 plays a key role in immune response and inflammation. Interleukin-1 receptor antagonist (IL-1RA) is a natural anti-inflammatory antagonist of IL-1, induces the expression of anti-inflammatory cytokines (like IL-10 and IL-4), and reduces the production of proinflammatory cytokines (like IFN-*γ*, IL-1, and IL-6) [17], reducing inflammation through competitive inhibition without triggering signaling cascades [18]. Imbalance of IL-1RA and IL-1 lead to the progression of various diseases [19]. It shows an association with various inflammatory and autoimmune diseases [20]. IL-1RA also owns an antiapoptotic effect in acute and chronic inflammation and can facilitate the proliferation of liver cell [21]. Serum IL-1RA concentration was negatively correlated with the end-stage liver disease score model, IL-1RA concentration, and IL-1RA/IL-1*β* ratio of HBV-ACLF patients in the death group was pronouncedly lower than those in the survival group [17]. This indicates that IL-1RA is vital in liver inflammation, and liver inflammation is relatively decreased in patients with HBV-ACLF, which is negatively correlated with the severity of the disease [17]. Above 96% of ACLF cases in China are associated with HBV [22]. It was found that levels of serum IL-1RA, IL-1*β*, IL-6, and TNF-*α* in patients with acute hepatitis B (AHB) and chronic hepatitis B (CHB) were lower compared to patients with HBV-ACLF [20, 22]. The ratio of IL-1RA to IL-1*β* was lower in the HBV-ACLF group compared with the AHB group [22], and the IL-1RA concentration and IL-1RA/IL-1*β* ratio of the death group were significantly lower than those of the survival group [22], but IL-1RA/IL-1*β* were higher, suggesting that endogenous IL-1RA alone was not enough to counter the action of IL-1, leading to cascade inflammation and liver injury [17], and the serum IL-1RA concentration was negatively correlated with the MELD score [22]. Studies have found that the serum IL-1RA level of female ACLF patients is obviously higher than that of males [17], and the IL-1RA produced by peripheral monocytes obtained from the female is 5-10 times that of male cells during the menstrual cycle [19]. Female ACLF patients may have a better prognosis than males. HBV-ACLF patients treated with IL-1RA may be a new idea.

NLRP3 is an important component of the inflammasome; it stimulates the maturation of precursors of the caspase1-dependent cytokines IL-1*β* and IL-18 (pre-IL-1b and pre-IL-18) [23]. NLRP3 inflammasome has been implicated in various liver diseases, and its activation has been implicated in liver injury. Low levels of NLRP3 are in normal liver tissue [24]. After LPS stimulation of monocytes, compared with healthy people, the expression of IL-1*β* in the liver tissue of ACLF patients was increased, while the level of IL-1*β* was decreased in advanced ACLF patients [25]. Among these single-nucleotide polymorphisms (SNP), two polymorphisms belonging to the IL-1 gene cluster were found (IL-1*β*:rs1143623 and IL-1ra:rs4251961), which are closely related to ACLF. Both SNPs are protective against ACLF, and these two polymorphisms in the IL-1 gene cluster appear to affect mortality in patients with decompensated cirrhosis, as 28-day survival in patients with combined protective SNP has seen an increase [26]. Wei et al. [27] conducted a meta-analysis on the relationship between IL-1*β*, TNF-*α* gene polymorphisms, and HBV infection, including 49 articles. The study found that 7 polymorphisms of IL-1*β* rs1143634 may be used as HBV infection potential genetic biomarkers. In addition, serum IL-1*α* was increased in patients with chronic hepatitis C (HCV), cirrhosis, and hepatocellular carcinoma (HCC) compared with healthy controls [28]. IL-1*β*-31 genotype is a biomarker for the development of cirrhosis and HCC susceptibility in patients with HCV [29]. In conclusion, high levels of IL-1 may be associated with poor prognosis of viral infection-associated liver diseases and HBV-ACLF.

## 3. Interleukin-6

As a kind of proinflammatory cytokine, IL-6 is a critical defense inducer of infection, and inflammation is more sensitive than CRP index [30, 31]. IL-6 is produced in monocytes, macrophages, T cells, fibroblasts, and endothelial cells, initiating the production of acute-phase proteins and rapidly activating the host defense system to perform multiple functions [32]. Studies have shown that compared with white blood cell (WBC) or C-reactive protein (CRP), IL-6 is a more sensitive marker for severe systemic inflammation in patients with severe liver injury, especially playing a key role in early liver regeneration response [33]. In addition, IL-6-mediated activation of STAT3 is a major driver of hepatocyte repair and replication, which promotes hepatocarcinogenesis. Elevated levels of IL-6 can be found in the serology of HCC patients [34].

The process of ACLF progress is closely related to the prognosis of systemic inflammation [35]. Studies have found that the IL-6 level of patients with HBV-ACLF rapidly progressing to death is higher than that of patients with survival, and IL-6 is an independent factor affecting the prognosis of HBV-ACLF. HBV-ACLF patients with high IL-6 levels have more than twice the risk of death compared to patients with low IL-6 levels [36]. However, it has been suggested that acute and only short-term increases in IL-6 levels may benefit liver regeneration, while chronic increases in IL-6 may adversely affect the liver and other organs. Some experimental studies have also shown that IL-6 is a cytokine with multiple biological functions, which can promote inflammatory response and maintain tissue homeostasis, and is associated with liver regeneration in animal models of acute liver failure. The complex function of IL-6 involves hepatic quasi- or transsignaling [37–39]. It can be seen that IL-6 level and duration simultaneously influence the development of HBV-ACLF [36].

Previous studies have demonstrated that plasma IL-6 increased with the increase of Th17 cells in patients with HBV-ACLF [40], and proinflammatory IL-6 is the main regulator of Th17 cell differentiation from naive T cells [41, 42]. IL-6 triggers signal transduction and activates intracellular STAT3 through dimerization of GP130 [41, 43]. We demonstrated that increased CD4^+^ T cell IL-6 expression is consistent with increased Th17 response in patients with HBV-ACLF [44], and IL-6 stimulation can activate the STAT3 pathway in peripheral blood mononuclear cells of patients with ACLF [44]. Th17 cells are a novel type of CD4^+^ T cell subset, which is the main mediator of tissue inflammation and is related to the pathogenesis of autoimmune and inflammatory diseases and also participates in the progression of liver failure [45]. Expressed in IL-6 and IL-17 patients with elevated ACLF immunohistochemical staining of liver tissue, the expression of STAT3 and mTOR rise [46]. And with mTOR, CD4^+^ T cells cannot be differentiated into Th17 cells; thus, the activation of IL-6 to STAT3 was reduced [46].

Hyper-interleukin-6 (HIL-6) is an artificial protein, including variants of IL-6 and glycoprotein 80 (soluble interleukin-6 receptor, sIL6R), linked by artificial short-linker [47]. Containing HIL-6 and hepatocyte growth factor (HGF) recombinant adenovirus Ad-HGF-HIL-6, compared with Ad-HGF or Ad-HIL-6, can significantly reduce the ACLF serum and tissue of high mobility group protein B1 (HMGB1) concentration and inflammation, and necrotic liver cells can be reduced, and ACLF can be more effectively protected in rat liver [47]. All of the above suggest that the protective mechanism of ACLF by AD-HGF-HIL-6 may be related to the HMGB1 signal, which deserves further study [47]. Wang et al. explored the association between IL-6 gene polymorphisms and susceptibility to liver disease through a meta-analysis that included 25 case-control studies, and patients with IL-6-specific genotype may have a higher risk of liver disease [48].

## 4. Interleukin-9 and Interleukin-10

IL-9 and IL-10 have been indirectly linked to HBV-ACLF progression. IL-9 is a cytokine produced by Th9 cells [49]. IL-9 and IL-10 levels were obviously lower in dead ACLF patients than in surviving ACLF patients, and baseline IL-9 levels indicated the prognosis of ACLF patients with 87.5% sensitivity and 61.5% specificity [49]. The expression of cytokines in the liver of ACLF patients is found to be unbalanced. Compared with CHB and healthy controls, the level of proinflammatory cytokines IFN-*γ* and TNF-*α* in the liver of ACLF patients is significantly upregulated [50], while the expression of TNF-*α* can be counterbalanced by IL-10. It has been reported that IL-10 treatment can normalize hepatitis C transaminase levels. Improve liver histology and reduce fibrosis [51]. Ye et al. included 22 case-control studies for meta-analysis and found that IL-10 rs1800871 polymorphism was associated with HBV risk, and allele C and genotype CC at IL10 rs1800871 locus may increase susceptibility to hepatitis B infection [52]. A meta-analysis showed that IL-10-1082GA gene polymorphism is associated with susceptibility to persistent HBV infection in the Chinese population, and IL-10-592CA gene polymorphism is associated with HBV clearance in the Chinese population [53]. In addition, increased IL-9 levels suggest failure of HCV sustained viral response in HCV infected patients [54]. IL-10 can be used as a new biomarker to evaluate the degree of inflammation in HCC development [55]. Compared with healthy controls, serum IL-10 was elevated in patients with HCV, cirrhosis, or HCC [56]. IL-10 RS180096 TT genotype, IL-10-592 CA polymorphism, and IL-10 ACC haplotype [56] are good markers for HCV patients to develop cirrhosis and HCC susceptibility. In conclusion, high levels of IL-10 may be associated with poor prognosis of viral infection-associated liver diseases, while the effect of IL-10 on HBV-ACLF remains to be studied.

## 5. Interleukin-12

IL-12 is secreted primarily by macrophages, which are key to initiating the cascade of immune-activated cytokines IFN-*γ*. Clinical studies have found that serum IL-12 in patients with HBV-ACLF is significantly elevated (approximately 2 times) compared with severe chronic hepatitis B, moderate chronic hepatitis B, and healthy controls [57, 58]. IL-12 induces IL-21 through transcription factors STAT1, STAT3, STAT4, and STAT5 [59] and enhances T follicular helper (Tfh) cells differentiation [60]. Upregulated Tfh cells and IL-21 are closely related to the severity and improvement of HBV-ACLF, and increased IL-12 may be related to the occurrence of ACLF. IL-12 promoted central memory CD8^+^ T cell response and functionally rescued the exhausted viral-specific CD8^+^ T cells in chronic HBV infection [61, 62]. Xue et al. showed that IL-12 levels significantly increased in patients with HBV-related liver failure compared with healthy controls [58]. In addition, IL-12 can be used as a new marker to assess the degree of inflammation in the development of HCC [55]. IL-12p40 levels are predictive markers of nonvirological response to HCV treatment with pegylated interferon and ribavirin in detecting treatment efficacy [63]. Thus, high levels of IL-12 may be associated with poor prognosis of viral infection-associated liver diseases, HCC and ACLF.

## 6. Interleukin-17

IL-17 is secreted by Th17 cells and stimulates CXCL8 production by hepatic stellate cells through the IL-17 receptor [64], which recruits immune cells to mediate large-scale tissue inflammation, thereby inducing severe liver inflammation [65]. CD4^+^ T cells produce IL-17 and participate in the pathogenesis of ACLF associated with HBV [44]. Memory T cells continuously produce IL-17, which may be one of the causes of progressive liver injury in ACLF patients^4451^. Data showed that Th17 cell level in HBV-ACLF patients or severe CHB patients was significantly higher than that in healthy people and CHB patients [40, 66, 67]. And the frequency of peripheral Th17 cells was correlated with the MELD score [68], and the ratio of Th17 to Treg cells were negatively correlated with the survival rate of ACLF patients [69]. Th17 cells preferentially produce IL-17A and IL-17F, with ROR*γ* T as the transcription factor in their differentiation [7, 70, 71]. Studies have shown that patients with ACLF ROR*γ* T express an increase. Therefore, ROR*γ* T may serve as a promising target for inhibiting Th17/IL-17-mediated inflammation in ACLF patients [72]. TMP778 inhibited ROR*γ* T-mediated Th17 cell activity [72], and the expression of IL-17A, IL-17F, IL-22, IL-23, IL-26, and CCR6. ACLF patients showed increased expression of CCR6 and IL-17R [73]. CCR6 is the hallmark chemokine receptor of Th17 cells and can promote the inflammatory function of Th17 cells [73].

It has been proven that the increased expression of IL-17 in HBV-ACLF disease is also closely related to the aggravation of CHB [40], the degree of liver fibrosis [74], and the severity of cirrhosis [75], indicating that IL-17 participates in the progression of HBV-ACLF disease. The expression of IL-17 can increase antiapoptotic molecules, improve the survival rate of virus-infected cells, and expand the persistent infection [76]. CD4^+^ T cells secreted IL-17A, which was not regulated by Treg cells, and reduced the levels of hepatitis B surface antigen (HBsAg) and hepatitis B envelope antigen (HBeAg) in the culture medium and hepatitis B virus DNA (HBV DNA) in HepG2.2.15 cells [69, 77]. The active resistance of IL-17A to various infections may amplify liver damage [74]. In terms of disease progression, elevated levels of IL-17 also indicate chronic progression to HCC in HCV patients [78], and IL-17 concentration is also a predictor of subsequent HCC development in patients with cirrhosis. The combination of AFP and IL-17 was very effective in predicting the incidence of HCC within 1 year [79].

Th17 cells were preferentially increased in ACLF patients and were positively correlated with Treg cells, and the increase of Treg cells in ACLF may be a result of the negative feedback effect of high Th17 [69]. The interaction between Treg cells and Th17 is key to sustaining the balance between immune response and pathological damage [69]. The ratio of Th17 to Treg cells frequency was significantly reduced in the HBV-ACLF survival group, and the ratio of most surviving patients was lower than 1.0 [69]. The ratio of Th17 to Treg cells may be a potential prognostic marker of ACLF. Research finds that in the IL-6 and IL-17 patients with increased ACLF immunohistochemical study of the liver tissue, the expression of STAT3 and mTOR increased [44, 46, 80]. Blocking mTOR can inhibit the differentiation of Th17 cells. mTOR can be used as a novel therapeutic target for patients with HBV-ACLF to inhibit Th17-mediated progressive liver injury [44].

## 7. Interleukin-18

IL-18 is constitutively expressed by natural killer-like B (NKB) cells and is stably secreted during infection [81]; NKB cells are a new immune subset isolated from NK cells and B cells. NKB cells have phenotypes of NK cells and B cells. NKB cells exhibit immunomodulatory functions in eliminating microbial infection and inflammation by secreting interleukin (IL)-12 and IL-18 [82]. IL-18 is a decisive factor in controlling Th1/Th2 balance during antiviral responses [83]. HBV X protein induces the expression of IL-18 in the liver and is closely related to liver damage during HBV infection [83]. IL-18 signaling induces the activation of two major pathways, including myeloid differentiation factor 88 (MyD88)/IL-1 receptor-related kinase/TNF receptor-related factor 6 and signal transducer and activator of transcription/mitogen activator kinases [84–86]. Both pathways ultimately mediate the phosphorylation of NF-*κ*B. The study found that low concentrations of IL-18 did not induce elevated NF-*κ*B phosphorylation, which may be due to insufficient neutralization of IL-18BP by IL-18. Conversely, high concentrations (10 ng/mL) of IL-18 might completely block the activity of IL-18BP and further increase phosphorylated NF-*κ*B, ultimately inducing NKB cell activity in HBV-ACLF, suggesting that in HBV-ACLF, IL-18 has potential positive feedback activity in regulating NKB cells during progression [82]. Clinical trials showed that compared with CHB, asymptomatic HBV carriers, and control group, the percentage of NKB cells in lymphocytes and the level of IL-18 in HBV-ACLF patients were significantly increased [82]. Elevated NKB cells and IL-18 may be important indicators of poor prognosis in patients with HBV-ACLF [82]. The increased proportion of NKB cells and the increased level of IL-18 in peripheral blood have good prognostic value on the survival status of HBV-ACLF. It indicates that IL-18 may be a hallmark cytokine secreted by NKB cells for regulating the inflammatory response of HBV-ACLF [82]. Studies show that IL-18-607A/C functional polymorphism is associated with susceptibility to an enhanced form of HBV DNA replication in chronic infection [83]. The -137C minor allele and the CG genotype are protective against chronic HBV infection [83]. Another study found that the -607A allele, -607AA, and -607AC genotypes were significantly higher in patients with high HBV DNA levels than those with low HBV DNA levels [83]. Yu et al. performed a meta-analysis of the relationship between IL-18 gene polymorphisms and viral hepatitis. The results demonstrate that the IL-18 rs1946518 and IL-18 rs187238 polymorphisms may be associated with the susceptibility of East Asians to HBV [87]. In addition, serum IL-18 levels were significantly increased as HBV disease progressed to HCC compared with controls [88]. In conclusion, IL-18 may be a biomarker for HBV related disease progression to HCC and ACLF.

## 8. Interleukin-21

IL-21 is a novel cytokine, which is produced mainly via activating CD4^+^ T cells and NK T cells. It can regulate the activation, proliferation, and survival of CD4^+^ T cells and B cells; the functional activity of CD8^+^ T cells and NK cells curb the differentiation of induced Treg cells, offset its inhibitory effect on effector T cells [89], stimulate T cell and B cell reactions, and regulate chronic viral infection [90]. On the other hand, IL-21 is likely to participate in liver injury by regulating the function of natural and adaptive immune cells and/or changing the expression of other inflammatory cytokines and can cause severe hepatitis through various ways [91]. Serum IL-21 levels in patients with severe chronic hepatitis B (S-CHB), moderate chronic hepatitis B (M-CHB), and healthy controls (HC) were lower compared to HBV-ACLF patients [57]. Hu et al. also found that serum IL-21 level and secretion increased in HBV-ACLF patients [91]. Previous research has shown the rs12508721T/C and rs2221903A/G polymorphisms of the IL-21 gene were associated with susceptibility to HBV-related HCC and chronic HBV infection [92]. The CD4^+^ Th cells secreting IL-21 increased in the HBV-ACLF group, while the CD4^+^ Th cells secreting IL-21 decreased in the HBV-ACLF recovered subjects, suggesting that CD4^+^ Th cells secreting IL-21 may participate in the pathogenesis of HBV-ACLF [91]. Upregulation of Tfh cells and IL-21 show a close association with the severity/improvement of HBV-ACLF. Serum of patients with HBV-ACLF rich in IL-21 enhances the differentiation of primary CD4^+^ T cells into Tfh cells, and this interaction can be blocked by anti-IL-21 antibodies [57]. The production of IL-21 was significantly correlated with the number of Tfh cells, clinical score, and liver function (ALT/AST), verifying the important role of IL-21 in Tfh cell generation during the occurrence of HBV-ACLF [57]. However, other studies have shown that there is no obvious correlation between the frequency of CD4^+^ IL-21 T cells and MELD score or survival rate of HBV-ACLF patients [91].

Similar to other cytokines that signal by a popular C-chain subunit, IL-21 activates Janus kinase (JAK)-a family of protein tyrosine kinases JAK1 and JAK3, and JAK1 binds to the IL-21 receptor (IL-21R), and JAK3 binds to the C-chain. IL-21R-induced signal transduction activates the signal transducers and transcriptional molecular activators [93]. In vitro experiments showed that IL21 upregulated IL-1*β*, IL-6, IL-10, IFN-*γ*, and TNF-*α* from peripheral blood mononuclear cell (PBMC). These cytokines are likely to be partially affected by IL-21 and may promote the development of HBV-ACLF [91].

## 9. Interleukin-22

IL-22, which was first discovered in 2000, was induced by IL-9 in thymic lymphoma, T cells, and other immune cells [94], belonging to the IL-10 cytokine family [95]. Human IL-22 is mainly produced by Th1 and Th22 T cell subsets, Th17 cells, and NK cell subsets [95]. IL-22 acts through a transmembrane receptor complex composed of IL-22R*α* and IL-10R2, which are expressed only in nonimmune cells, including hepatocytes, renal cells, keratinocytes, intestinal, or respiratory epithelial cells [95–97]. IL-22 is transmitted through JAK and STAT signaling pathways by binding dimer IL-22R and activates transcription factors STAT1, STAT3, STAT5, etc., through phosphorylated kinases Jak1 and Tyk2 [95, 98]. Participate in inflammation and damage of different tissues [97, 99]. IL-22 has a proinflammatory function and is likely to damage the prognosis of severe liver patients. Studies have found that in patients with cirrhosis, high serum IL-22 concentration is associated with the occurrence, progression, and mortality of ACLF [100]. Circulating IL-22 was associated with MELD score and the chronic liver failure consortium (CLIF-C) acute-on-chronic liver failure score (CLIF-C ACLFs). Elevated levels of plasma and intrahepatic IL-22 in patients with HBV-ACLF are about 1.3 times that of patients with CHB [101], and increased circulating IL-22 is related to a low survival rate of HBV-ACLF [101]. In vitro studies have shown that IL-22 promotes liver inflammation via Th17 cells and macrophages to the liver in HBV-infected people and mice and induces the production of proinflammatory mediators and acute phase proteins in liver cells [102]. Persistent intrahepatic inflammation may result from insufficient host immunity to clear the viral infection, leading to the development and progression of HBV-ACLF. Moreover, proliferative and antiapoptotic activities of IL-22 may accelerate the growth of existing HCC [103]. IL-22 binding protein (IL-22BP) is a soluble inhibitor of IL-22 signal transduction and can prevent il-22 from binding to its transmembrane receptor, which is a single chain receptor of IL-22. The study found that patients with ACLF of IL-22BP/IL-22 ratio is lower than the patients without ACLF [100], during the period of cirrhosis development for ACLF and eventually death rates of IL-22BP/IL-22 which are declining gradually [100]. In vitro experiments have shown that IL-22BP can inhibit IL-22 signal transduction in hepatocytes and inhibit the generation of proteins in the acute phase [100]. High levels of IL-22BP can neutralize IL-22 in vitro and have protective effects in mouse acute liver failure models [104].

Studies have demonstrated that IL-22 advanced the expansion of liver progenitor cells/stem cells in mice and hepatitis B patients in a STAT3-dependent manner, showing a role in promoting liver regeneration [105]. In animal models of concanavalin-induced hepatitis or alcoholic hepatitis, the application of IL-22 can reduce the severity of liver disease [106, 107]. By creating a severe liver injury model using serum and liver samples from patients with ACLF, IL-22 therapy was subsequently demonstrated to improve the survival of ACLF by alleviating bacterial infection [108]. In mice, IL-22 prevents hepatocytes from apoptosis in a Mir-15A/16-1-dependent manner [109]. The liver protection or liver damage shown by IL-22 in different liver diseases may be due to the stage dependence (i.e., acute and chronic) and/or disease dependence of IL-22.

## 10. Interleukin-23

IL-23 is from macrophages and dendritic cells (DCS) and exerts a key role in bridging congenital and adaptive immune responses [110]. Serum IL-23 level in patients with CHB is positively correlated with liver injury [111]. HBV infection induces the production of IL-23 via antigen-presenting cells and causes liver injury through the IL-23/IL-17 axis [112]. Persistent IL-23 generation of liver inflammatory macrophages responding to damaged hepatocytes after chronic HBV infection altered macrophage function for HCC promotion. Blocking IL-23 activity may be benefited CHB patients who had a high risk of HCC [113]. Serum IL-23 was significantly upregulated in CHB and ACLF patients, and the upregulated IL-23 was significantly correlated with the aggravation of HBV-ACLF, and the serum IL-23 level in HBV-ACLF patients after treatment was significantly lower than that in the nonsurvival group [114]. There was a significant positive correlation between IL-23 expression and INR/PT/MELD score system in HBV-ACLF patients, and there was a positive correlation between IL-23P19 mRNA expression and TBIL. Elevated IL-23 cytokine levels have been observed in single-cell-derived dendritic cells from patients with HBV-ACLF, which is associated with high mortality [114].

IL-23R expression increase is related to the severity of acute or chronic liver failure [115]. IL-23R was not only pronouncedly correlated with alanine aminotransferase (ALT), straight bilirubin (SBil), Child-Turcotte-Pugh, and MELD scores but liver cirrhosis. IL-23R is critical to the production of pathogenic Th17 cells [115], and ACLF patients expressing high IL-23R on Th17 cells lead to the activation of the STAT3 pathway and the functional activation and maturation of Th17 cells [116], which can induce inflammation and is closely related to the severity of liver disease [115]. Specifically, targeted inhibition of IL-23-induced STAT3 phosphorylation can reduce liver inflammation [117]. Another study showed that the IL-23R R381Q gene variant prevents immune-mediated disease through blocking the IL-23-induced effector response of Th17 cells [118]. Comprehensive to these results, we conclude that Th17 cells increased in patients with ACLF IL-23R expression in peripheral blood and liver, and Th17 cells produce a large number of inflammatory cytokines and cause severe inflammation and disease progression of the pathogenic microenvironment. The NF-*κ*B pathway is likely to participate in the expression of IL-23 in HBV-ACLF patients [114]. The expression of NF-*κ* BP65 was elevated in HBV-ACLF patients, suggesting that IL-23 and NF-*κ*B signaling pathway may lead to poor prognosis of HBV-ACLF.

## 11. Interleukin-27

IL-27 belongs to the IL-12 family as a heterodimer somatic factor consisted of two subunits of EPeb virus inducible gene 3 (EBI3) and IL-27P28 [119], binding to receptors with GP130 and IL-27RA to activate JAK-STAT and MAPK signaling pathways [120]. IL-27 can play a proinflammatory and anti-inflammatory role during immune response [117, 120]. IL-27 supports antibody-driven autoimmune diseases by affecting B cells [121, 122]. Studies have found that serum IgG, IgA, and IgM levels of HBV-infected patients are positively correlated with serum TBil levels and negatively with prothrombin activity (PTA) and albumin levels, which are commonly used as markers of liver injury [123]. IL-27 is positively correlated with the level of immunoglobulin in vivo, indicating that IL-27 facilitates the induce of immunoglobulin and aggravates liver injury. High levels of IL-27EBI3 cytokine or IL-27RA expression are associated with poor prognosis in HCC patients. We suspect that a higher level of IL-27 may be a marker to predict the poor prognosis of HBV-ACLF [36, 57, 124].

## 12. Interleukin-31

IL-31, a newly discovered proinflammatory cytokine [125], is mainly produced by CD4^+^ T cells and regulated by the JAK-STAT, PI3K/AKT, and RAS/ERK signaling pathways [126, 127]. It has been demonstrated and shown that serum levels of TGF-*β*1 and IL-31 are significantly enhanced in ACLF patients, and the TGF-*β*1/IL-31 pathway is likely to contribute to progressive liver injury in ACLF through direct inflammatory function and expression of other inflammatory cytokines that regulate innate and adaptive immune cells [112]. TGF-*β*1/IL-31 pathway displayed high sensitivity and specificity in the prediction of ACLF nonsurvivors (85.7% and 100.0%, respectively) [112]. Moreover, TGF-*β*1 and IL-31 are associated with the progression of chronic hepatitis B to cirrhosis and are closely related to the severity of hepatitis B virus-related liver cirrhosis (HBV-LC). In patients with HBV-related cirrhosis, serum TGF-*β*1 and IL-31 were significantly elevated, with sensitivity and specificity of 90.9% and 66.7%, respectively. These findings suggest a possible role of the TGF-*β*1/IL-31 pathway in the pathogenesis of liver fibrosis during chronic hepatitis B virus infection [128]. TGF-*β*1 appears to bind to the function of IL-31. TGF-*β*1 is a 25kDa homologous dimer protein, which consists of two subunits connected via disulfide bonds and is a strong inhibitor of DNA synthesis and cell proliferation [129]. Miwa et al. found that TGF-*β*1 was obviously increased in both plasma and liver tissue of patients with fulminant liver failure (FLF) [130]. Besides, TGF-*β*1 curbed liver regeneration and facilitated perineural fibrosis and hepatocyte apoptosis in fulminant hepatic failure (FLF) rat models [131]. The biological function of TGF-*β*1 relies on signal transduction and Smad protein regulation. TGF-*β*1 was found to promote Smad2 phosphorylation and the binding of Smad3 to the IL-31 promoter and then the IL-31-JAK-STAT signaling pathway [132].

## 13. Interleukin-33

ACLF patients usually present with monocyte dysfunction and excessive systemic inflammatory response [133]. Serum interleukin-33 (IL-33) levels are correlated with the severity of the liver disease. Meanwhile, IL-33 is expressed in both cancer cells and stromal cells in the HCC microenvironment, which may be a key tumor promoter for HCC proliferation and tumorigenicity and negatively correlates with survival of HCC patients [134, 135]. IL-33 regulates immune response as a risk pattern (DAMP)-related molecule [133]. Compared with patients with chronic hepatitis B and the control group, the expression level of IL-33 in peripheral blood and liver of patients with HBV-ACLF was significantly increased [133]. It was found that the expression of HLA-DR, CCR2, and CD80 was significantly increased in monocytes treated with IL-33. In a patient with HBV-ACLF, IL-33 enhances LPS-induced monocyte inflammatory storm through ERK1/2 activation, and the systemic inflammatory storm is the main driver of ACLF, leading to over activation of the innate immune system [133]. Therefore, high levels of IL-33 are a marker of the poor prognosis of ACLF.

## 14. Interleukin-35

IL-35 belongs to IL-12 members of the family of IL-12 [136] and is mainly secreted by CD4^+^ T and CD8^+^ T regulatory Treg cells, activated dendritic cells, and regulatory B cells and has shown immunosuppressive function in various infectious diseases, cancers, and autoimmune diseases [137–139]. In tumor tissues of patients with HBV-related HCC, high expression of IL-35 was associated with tumor cell invasion and poor prognosis. It is also an independent prognostic factor for HCC recurrence [140, 141]. During the period of acute and chronic viral hepatitis, IL-35 immunosuppression functions mainly through promoting the proliferation and differentiation and CD8^+^ T cell function and inhibition of Th17 Treg cells T cell toxicity; CD8^+^ T cell-mediated cytotoxicity mechanisms include the direct cytolytic activity of target cells and noncytolytic activity of cytokine-mediated tissue damage [141–148].

**Figure 1:**
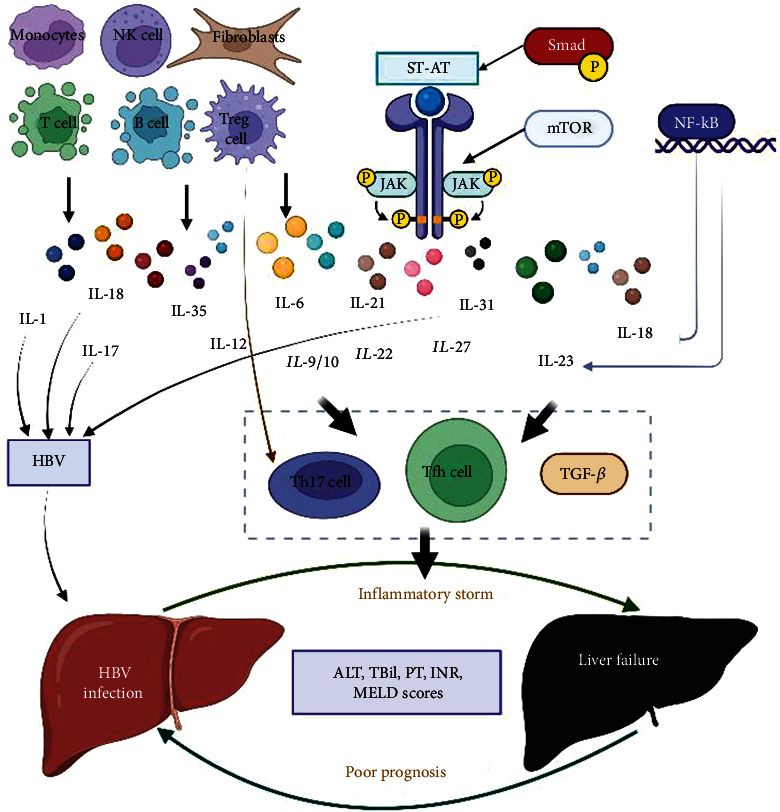
Effects of ILs on the prognosis of HBV-ACLF and its mechanism. (Illustration: the picture shows the main cells producing ILs; the key mechanisms of different ILs affecting the prognosis of ACLF; the key signaling pathways that can regulate the function of ILs, and the key cytokines that cause the inflammatory storm of liver and summarized the whole paper briefly.)

ACLF patients have a complex immune state, accompanied by excessive inflammation and immune failure [149]. Compared with patients with chronic viral hepatitis and healthy people, ACLF patients with viral hepatitis induced increased serum IL-35 level [150], which was positively correlated with total bilirubin and negatively with prothrombin time activity [150], indicating a possible relationship between IL-35 and severe liver injury [150]. In patients with ACLF, IL-35 stimulus inhibiting nonspecific antigen cytotoxic CD8^+^ T cell activity and inhibiting cytokines mediated target cell death, suggesting IL-35 to CD8^+^ T cell immune inhibition. CD8^+^ T cells in ACLF patients showed depletion phenotype, and the expression of perforin, granzyme B, and FasL mRNA in peripheral CD8^+^ T cells was downregulated. Immune checkpoint molecules (PD-1, CTLA-4, and LAG3) were increased. Increased IL-35 expression in ACLF patients may lead to T cell dysfunction or failure by reducing the induction of cytotoxic and immune checkpoint molecules in ACLF patients which leads to the poor prognosis. They are shown in Figure 1.

## 15. Conclusion

Among the above, more than ten kinds of ILs, IL-1, IL-6, IL-12, IL-17, IL-18, IL-21, IL-23, IL-33, IL-31, and IL-35 may be the prognostic indicators for HBV-ACLF. Among them, IL-1, IL-6, IL-17, IL21, IL-23, and IL-35 are relatively important regulatory factors, which may affect systemic inflammatory response due to their own changes along with changes in other ILs levels.

The mechanism of ILs affects the prognosis of ACLF and can be summarized as the following categories: (1) the levels of ILs are closely associated with HBV infection, which affect the degree of liver inflammation in patients with HBV, aggravating liver damage and leading to the occurrence and bad prognosis of ACLF patients (e.g., IL-1, IL-17, IL-18, and IL-31); (2) affecting the functions of CD4^+^ T, CD8^+^ T, and Treg cells may lead to the immune dysfunction or failure of systemic T cells and result in poor prognosis of ACLF patients (e.g., IL-17, IL-21, IL-22,IL-31, and IL-35); and (3) further studies indicated that the STAT signaling pathway (IL-6, IL22, IL23, IL27, and IL31), JAK signaling pathway (IL-21, IL-22, and IL-31), mTOR signaling pathway (IL-6 and IL17), NF-*κ*B signaling pathway (IL18 and IL-23), and Smad signaling pathway (IL-31) may regulate the expression of related ILs. Thus, the expression of many cytokines such as IFN-*γ*, TNF-*α*, and TGF-*β* can be affected, and the immune status and prognosis of ACLF patients can be affected. For example, the activation of the key signal pathway JAK/STAT signal pathway can promote the activation of Th17 cells and Tfh cells by upregulating the expression of IL-21 and IL23, aggravating the inflammatory storm of liver, and leading to the occurrence and development of ACLF. The JAK/STAT signaling pathway can be activated by phosphorylation of Smad, which promotes the production of TGF-*β* and IL31, inhibits hepatocyte regeneration, and promotes ACLF development. Activation of the mTOR/STAT signaling pathway can inhibit CD4^+^ T cells to differentiate into Th17 cells, thus inhibiting Th17-mediated progressive liver injury and improving the prognosis of ACLF. In addition, the clear correlation between ALT, TBil, PT, INR, and MELD scores and ILs also suggest that ILs can reflect the severity of liver disease and the prognosis of HBV-ACLF to a certain extent. However, the predictive value of the above-mentioned ILs needs further study and verification.

## Data Availability

Figure 1 has been uploaded in PDF version, and we provide the mapping web site: https://app.biorender.com/illustrations/62c2e2a4d3bae23d89164996. After logging in the web, the picture will become editable state.
